# On-chip measurements of protein unfolding from direct observations of micron-scale diffusion†
†Electronic supplementary information (ESI) available. See DOI: 10.1039/c7sc04331g


**DOI:** 10.1039/c7sc04331g

**Published:** 2018-02-09

**Authors:** Yuewen Zhang, Emma V. Yates, Liu Hong, Kadi L. Saar, Georg Meisl, Christopher M. Dobson, Tuomas P. J. Knowles

**Affiliations:** a Department of Chemistry , University of Cambridge , Lensfield Road , Cambridge , CB2 1EW , UK . Email: cmd44@cam.ac.uk ; Email: tpjk2@cam.ac.uk ; Tel: +44 (0)1223 336344; b Zhou Pei-Yuan Center for Applied Mathematics , Tsinghua University , Beijing , 10084 , P. R. China; c Cavendish Laboratory , University of Cambridge , J J Thomson Avenue , Cambridge , CB3 0HE , UK

## Abstract

Investigations of protein folding, unfolding and stability are critical for the understanding of the molecular basis of biological structure and function. We describe here a microfluidic approach to probe the unfolding of unlabelled protein molecules in microliter volumes. We achieve this objective through the use of a microfluidic platform, which allows the changes in molecular diffusivity upon folding and unfolding to be detected directly. We illustrate this approach by monitoring the unfolding of bovine serum albumin in solution as a function of pH. These results show the viability of probing protein stability on chip in small volumes.

## Abstract 2

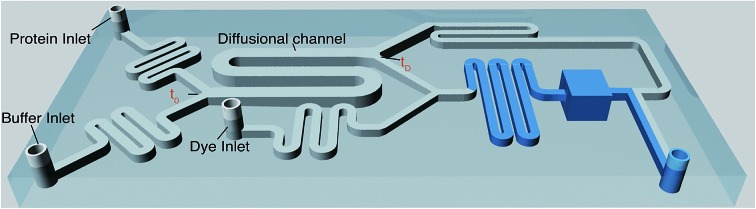
The unfolding process of BSA in solution as a function of pH was studied by microfluidic diffusional sizing device.

## Introduction

Biomolecular stability plays an important role in virtually every biological process taking place within living systems. Specifically, to perform their diverse biological functions, most proteins must fold precisely into their unique three-dimensional native structures. Incorrect protein folding will often cause malfunction, and can give rise to a range of human diseases.^1^–^6^ Indeed, a particularly prevalent class of disorders associated with the aberrant folding of proteins involves amyloid formation and is connected to neurodegenerative diseases, such as Alzheimer's disease, Huntington's disease and Parkinson's disease.^2^,^7^,^8^

Proteins can be denatured by changing their chemical or physical environments, such as adding chemical denaturants, changing the solution pH value, heating or applying pressure. The thermodynamic stability of the folded state of proteins, quantified as the Gibbs free energy difference between the folded and unfolded states, is commonly probed through denaturation experiments, which promote unfolding.^9^ A number of methods have been established for studying the unfolding of protein structures, including circular dichroism (CD),^10^ nuclear magnetic resonance (NMR) spectroscopy,^11^–^13^ dual polarisation interferometry (DPI)^14^ and fluorescence-based optical techniques.^15^ These methods have advanced very significantly our understanding of the nature of protein structure and stability. Generally, however, these approaches require high concentrations of protein, need long processing times of several hours, and may cause changes in the native folded protein structure due to the installation of labels which are often used to enhance optical or magnetic signals.^16^

Micron-scale measurements of molecular diffusivity have been shown to be a highly sensitive approach to define the sizes of proteins and to bring together the benefits of label-based and label-free methods.^17^–^19^ The ability to assess rapidly the folding state of a protein, using small volumes of unlabelled analytes, could have applications for laboratory scale protein science, where stability is a key parameter of interest, as well as for personalized medicine and diagnostics. Indeed, a commonly used modality to detect the binding of small molecule drugs to protein targets is to follow the resultant increase in the stability of the native state, a process which could be miniaturised using platforms of the type described in this paper. Microfluidic systems are highly portable, cost effective, and can easily be integrated into sensing platforms with potential applications in personalized medicine.^20^,^21^ Recently, we reported a microfluidic approach for measuring the sizes of proteins^18^ with the key characteristics that the proteins of interested are labelled on-chip with a fluorogenic dye immediately prior to an optical detection step. This approach has the additional advantage of allowing the study of proteins under well defined conditions and with highly sensitive detection.

In this study, we set out to explore how this microfluidic diffusional sizing (MDS) approach can be used to study the changes in protein size induced by folding and unfolding. In particular, this approach was employed to study in detail the denaturation of bovine serum albumin (BSA) induced by varying the pH of the solvent.

## Results and discussion

**Fig. 1:**
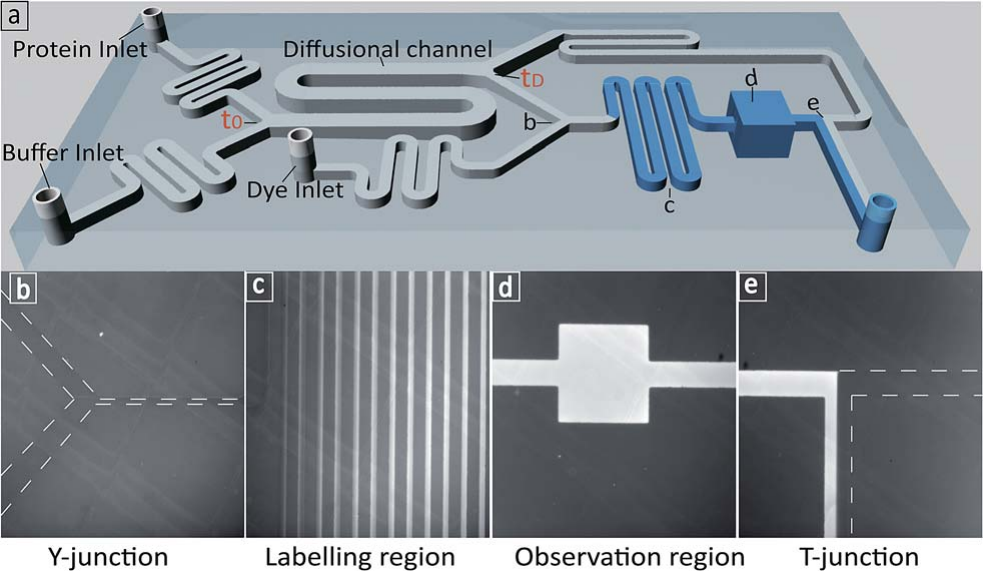
(a) 3D schematic of the microfluidic diffusional sizing (MDS) device used in this study.^18^ (b) The Y-junction showing the protein mixing with fluorogenic labelling solution after the diffusion step. (c) The labelling region for *o*-phthalaldehyde (OPA) react on-chip with primary amine containing residues on the protein.^24^–^27^ (d) The observation region for monitoring the fluorescence intensity of the labelled protein. (e) The T-junction showing the flow of labelled protein and unlabelled protein solution.

We first set out to measure the molecular diffusivity of BSA in its folded and unfolded states on-chip. The architecture of the microfluidic diffusional sizing (MDS) device^18^ is shown in Fig. 1a. Briefly, the protein and buffer streams mix at the position labelled *t*_0_. At this point, the protein molecules have not diffused into the buffer stream, and they have the same initial distribution irrespective of molecular weight or structure. Each stream spans half the width of the diffusional channel (Fig. 1a and 3a), and has equal volumetric flow rates. The protein molecules then diffuse laterally into the buffer stream as the solution flows through the diffusional channel. At the end of the diffusional channel (*t*_D_), the proteins of smallest hydrodynamic radius (*R*_h_) have diffused furthest into the buffer stream. Subsequently, a third of the total stream (Fig. 1a and 3a) is diverted into the latent labelling region (Fig. 1c), and the diffused protein molecules are quantitatively labelled with a fluorogenic dye. In the diffusional channel, the mixing process proceeds exclusively *via* diffusion as convective mixing is suppressed in small volumes of low Reynolds numbers.^22^ The total concentration of protein molecules diverted for labelling is therefore determined by the diffusivity alone and hence by the protein *R*_h_.^18^ Thus, measuring the fluorescence intensity in the observation region (Fig. 1d) defines the total concentration of protein diverted for labelling at position *t*_D_, which in turn reveals the protein distribution at position *t*_D_ allowing determination of *R*_h_, by comparison with values simulated for the diffusion of particles of known *R*_h_ values.^18^,^23^

**Fig. 2:**
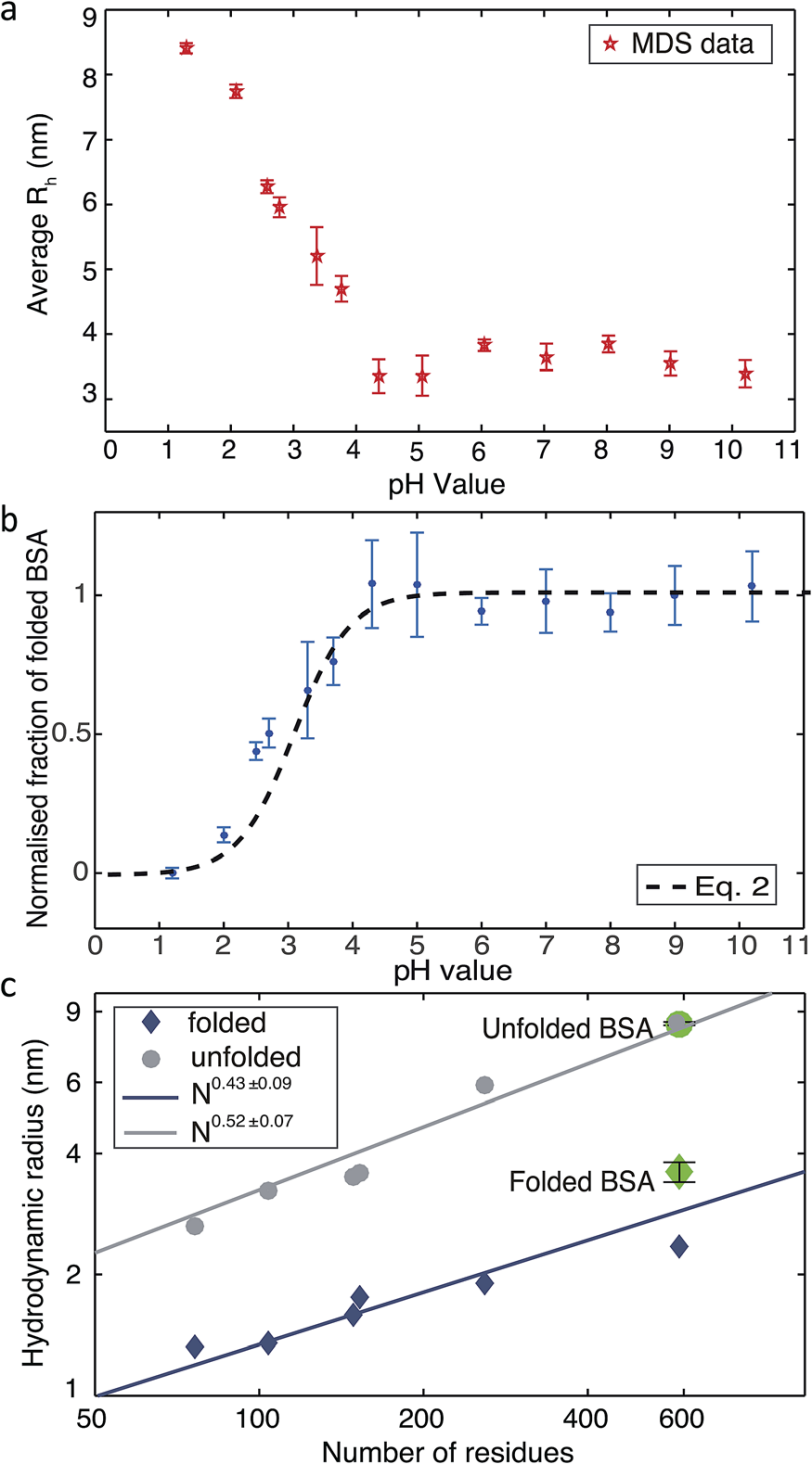
(a) The average *R*_h_ of BSA measured by the MDS device in buffer solutions of varying pH. (b) The normalized fraction of folded BSA derived from the measured *R*_h_. (c) Plots of the average *R*_h_*versus* the number of residues in a polypeptide chain. The values for folded and unfolded BSA (shown in green) were measured using the MDS device. Literature values are shown as blue diamonds and grey circles for a range of folded and unfolded proteins respectively.^13^,^30^–^36^

Changing the pH is a common way to achieve protein denaturation. The reason for protein denaturation under varying pH conditions is that some buried ionizable groups of side chains within the polypeptide sequence have a highly perturbed p*K*_a_. Typically, the buried groups of proteins have lower pK_a_ values in the native state than in the denatured state.^9^ This difference creates a thermodynamic driving force increasingly favouring the unfolded state when the pH of the solution is lowered. In order to probe the unfolding process, we measured the average *R*_h_ values under different pH conditions. Solutions of BSA and buffers adjusted to pH values between 1.2 to 10.2 were injected into the ‘protein inlet’ and the ‘buffer inlet’ on the MDS device, respectively (Fig. 1a). After diffusional mixing, the BSA molecules that had diffused across at least one-sixth of the diffusional channel width were diverted for labelling on chip, prior to detection *via* fluorescence emission in the observation region (Fig. 1d). Then, fluorescence intensities in the observation region were compared between the case when the protein sample has diffused and that obtained for a homogeneous distribution achieved by injecting the protein sample into both inlets. Thus, by comparing observed fluorescence intensities with the simulated values for the diffusion of particles of known *R*_h_, the average *R*_h_ of the protein was calculated.^18^ As shown in Fig. 2a, the average *R*_h_ of BSA is almost constant when the pH of the buffer is between 4.3 and 10.2; at pH 7.0, the average *R*_h_ for BSA was found to be 3.60 ± 0.41 nm, which is consistent with the value of 3.39 ± 0.27 nm measured by fluorescence-based techniques.^28^ The structure of BSA is therefore folded between pH 4.3 and pH 10.2. When the buffer pH was reduced below 4.3, the average *R*_h_ value was observed to increase progressively (Fig. 2a), consistent with the unfolding of the protein, and at pH 1.2, the average *R*_h_ value is 8.4 ± 0.16 nm. Based on the measured average *R*_h_ of BSA and linear interpolation *R*_h_ = (*R*maxh – *R*minh) × (1 – *f*_N_) + *R*minh, the fraction of folded BSA is calculated (Fig. 2b). The average *R*_h_ values for BSA measured using the MDS device fits well to a polymer scaling law^29^ between hydrodynamic radius and number of residues (*R*_h_ ∝ *N*^α^, Fig. 2c).

**Fig. 3:**
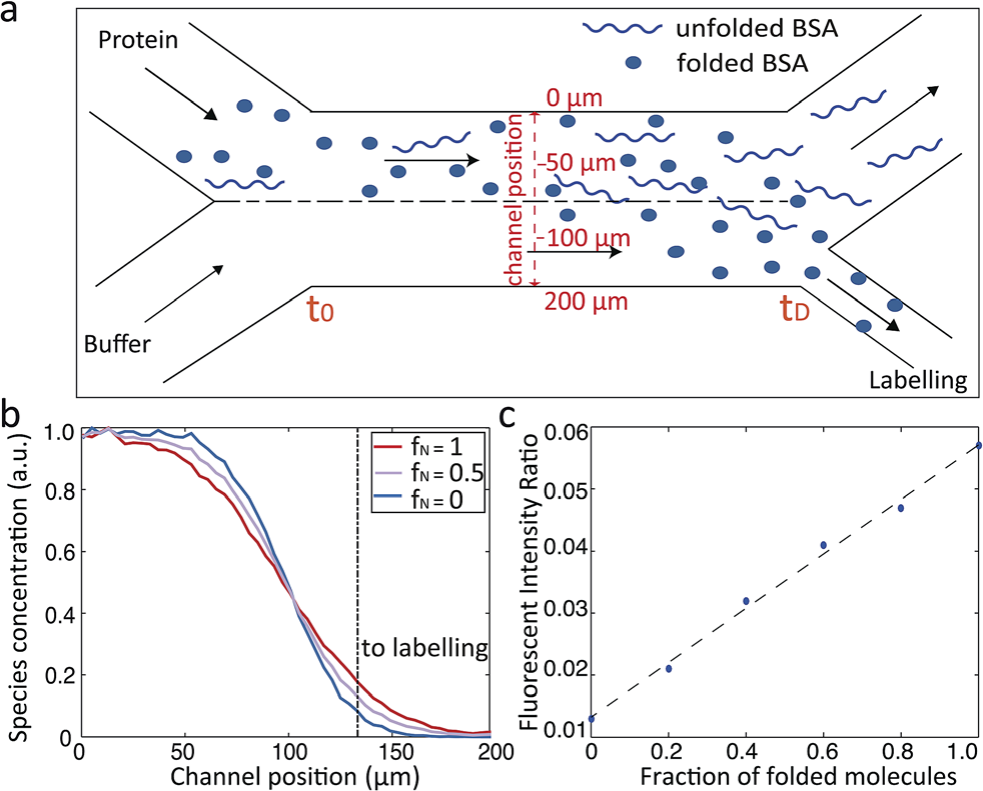
(a) Schematic illustration of BSA diffusive process within the diffusional channel of the MDS device. (b) Diffusive behaviour of a mixture of folded and unfolded BSA at the end (*t*_D_) of the diffusional channel. Completely folded and completely unfolded BSA correspond to *f*_N_ = 1 and *f*_N_ = 0 respectively. 50% folded and 50% unfolded BSA corresponds to *f*_N_ = 0.5. (c) Calibration curve of the fraction of folded and unfolded BSA against the fluorescence intensity ratio.

The microfluidic approach can be used not only to obtain the average *R*_h_ value of folded and unfolded BSA, but also to derive the relative populations of the two forms in a given solution. We set out to use this approach to elucidate the relative proportions of folded and unfolded BSA at each of the different pH values for which we had experimentally obtained the average *R*_h_ (Fig. 2a). With a mixture of folded and unfolded BSA molecules, we expect the more compact folded BSA to diffuse further across the microfluidic diffusional channel towards the labelling region than the unfolded BSA. Therefore, a more intense fluorescence signals will be measured in the observation region for the sample with larger relative proportion of the folded BSA (Fig. 3a).

We simulated the behaviour of the system containing different populations of folded (3.5 nm) and unfolded (8.4 nm) BSA molecule in a rectangular channel 200 μm in width, 25 μm in height and 17 000 μm in length at a flow rate of 25 μL h^–1^, as was used in the measurements.^18^ The simulations were based on solving the Langevin equation describing diffusion advection behaviour.^23^,^37^–^39^ For each ratio of folded and unfolded BSA, the diffusion was simulated as follows: one with half of the channel filled with the protein molecules and the other with the full channel filled in order to match the experimental protocol. The distributions of the typical binary BSA mixtures determined at the end of the diffusional channel (*t*_D_) are shown in Fig. 3b. From these profiles for each of the simulations, we extracted the number of molecules that had diffused far enough to enter the fluid stream that flows into the labelling region of the device (Fig. 3b dotted line). By comparing the relative intensities of the two simulations, we constructed a calibration curve that linked the recorded fluorescence intensity ratios to the fraction of folded and unfolded protein molecules (Fig. 3c). This constructed curve was then used to relate the observed fluorescence intensities at each of the pH values and the average *R*_h_ to the relative population of folded and unfolded proteins in the mixture. The obtained unfolding curves agree well with previously published results of acid unfolding measured by different techniques.^40^

**Fig. 4:**
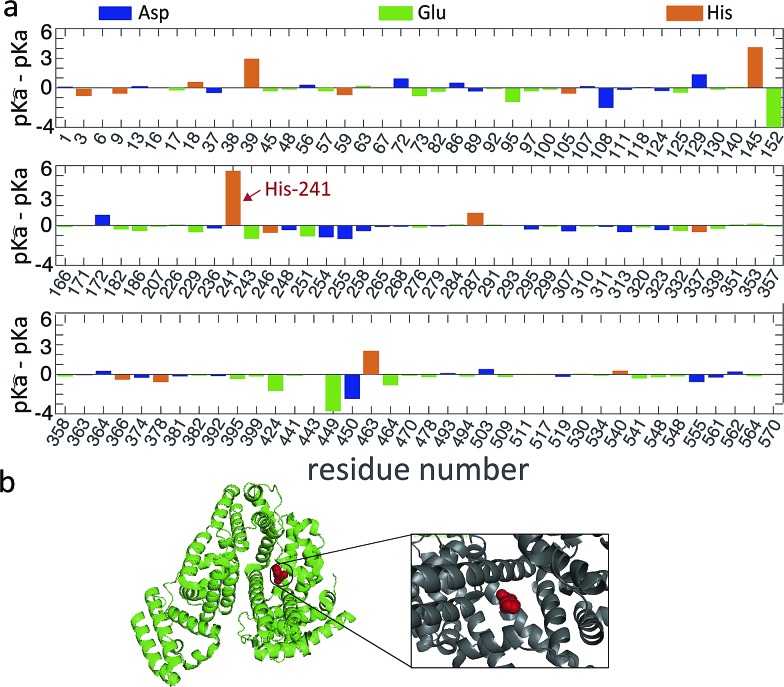
(a) The difference in p*K*_a_ values between folded and unfolded states for the Asp, Glu and His residues of BSA along the sequence. The key titration site His241 is indicated by an arrow. (b) The crystal structure of BSA (PDB ID: ; 4F5S) is shown in ribbon structure. His241 (red sphere) is highlighted and shown in more detail in the inset.

To obtain an estimate for the unfolding free energy, we explore whether a single ionizable group can act as a key titration site during the unfolding process (*m* = 1). Indeed, analysis of the pK_a_ values of the ionizable residues of the BSA sequence reveals that one particular residue, His241, has large difference between its p*K*_a_ values in the folded and unfolded states (Fig. 4a).

More specifically, since the pH induced unfolding transition observed in Fig. 2a occurs between pH = 1.2 and pH = 4.3, the p*K*a values for the potential titration sites must span this interval. This condition guarantees that over the pH range (from pH 1.2 to 4.3), where BSA is observed to unfold, the ionizable groups in the unfolded state is increasingly protonated, while the folded state remains unchanged, thus driving the equilibrium towards the unfolded state (see ESI† for details). In particular, for a single titration site *m* = 1 and given that the unfolding occurs over the pH range [1.2, 4.3], we require p*K*_a_ < 1.2, p*K*_a_ > 4.3 and hence p*K*_a_–p*K*_a_ > 3.1. The p*K*_a_ values of each ionizable group in the folded state were predicted by computational analysis (DEPTH server,^41^Fig. 4a), leading to the identification of one key titration site, His241, satisfying the above criterion with p*K*_a_ = 6.04 and p*K*_a_ = 0.6. The p*K*_a_ values for other residues (such as Glu and Asp) do not meet the requirement (Fig. 4a). For single titration site, thermodynamic arguments (eqn (S6)†) yield a simple expression for the fraction of folded protein.1
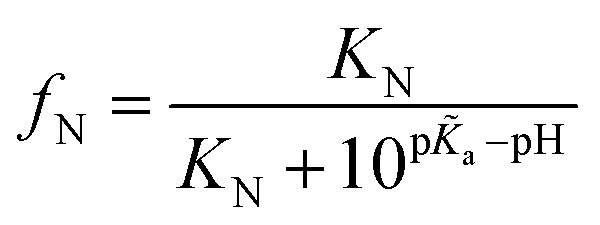
where *f*_N_ is fraction of folded protein; *K*_N_ is the equilibrium constant for BSA folding at the given pH. Based on the microfluidic results, *K*_N_ = 2213 ± 272, corresponding to a standard free energy Δ*G*^Θ^ = –4.55 ± 0.08 kcal mol^–1^ (see ESI† for details).

We also investigated the extent of secondary structure change under different pH conditions by circular dichroism (CD) spectroscopy. The normalized fractions of folded BSA derived from the molar ellipticity at 222 nm, 208 nm and total integrated area between 200–250 nm were calculated using eqn (S2) and (S3) (Fig. S2†). From the CD spectra, the folding free energy of the BSA was determined to be Δ*G*^Θ^= –4.24 ± 0.03 kcal mol^–1^ (see ESI† for details), which agrees well with the value of –4.55 ± 0.08 kcal mol^–1^ based on our microfluidic results, as well as –4.04 kcal mol^–1^ and –4.60 kcal mol^–1^ reported from previous studies.^42^ This close agreement of the free energies obtained in our study by unfolding at neutral pH *via* denaturant, suggests that any protonation of residues other than His241 at pHs above 4.3 does not significantly affect the relative stabilities of the native and unfolded states.

**Fig. 5:**
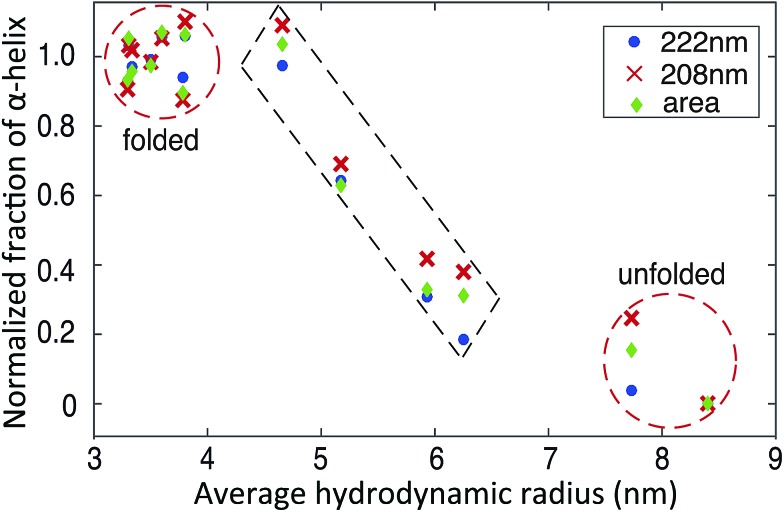
Plots of the average *R*_h_*versus* the normalized fraction of α-helix shows clustering in different states. The normalized fraction of α-helix derived from the molar ellipticity at 222 nm, 208 nm and total area between 200–250 nm are calculated using eqn (S2).†

The availability of both microfluidic and CD measurements allow us to carry out multi-dimensional cluster analyses of the folding and unfolding process (Fig. 5). We observe two major clusters, corresponding to the folded and unfolded state, in agreement with the reported two-state folding behaviour of BSA.^42^ Interestingly, in addition four data sets with intermediate average *R*_h_ values and α-helix contents are also observed which correspond to mixtures of folded and unfolded BSA, identifying the trajectory from the folded to unfolded state (Fig. 5). The fact that the folding transition occurs in a similar manner along a coordinate measuring global structure (*R*_h_, horizontal axis, Fig. 5) and along a coordinate sensitive to local structure (fraction of α-helix, vertical axis, Fig. 5) supports the two-state nature of this transition.

## Conclusions

In this work, we have shown that our MDS approach can be used to investigate the process of protein unfolding. In particular, by measuring the average *R*_h_ of BSA, which is unfolded under acidic conditions (between pH 1.2 and 4.3). The average *R*_h_ for both folded and unfolded BSA were measured to be 3.6 ± 0.41 nm and 8.4 ± 0.16 nm, respectively. During the unfolding process, the relative fractions of folded and unfolded BSA were calculated based on the measured average *R*_h_ of a two-state model.

Compared to conventional techniques this approach uses significantly less sample with the MDS device only requiring microliters of sample solution. The residence time is in the order of a few seconds for each measurement. We therefore anticipate that this microfluidic approach will open up new possibilities for the study of the structural stability of proteins and other biomolecules under a variety of conditions.

## Conflicts of interest

There are no conflicts to declare.

## Supplementary Material

Supplementary information
