# Supplementary material for: On-chip measurements of protein unfolding from direct observations of micron-scale diffusion
Source: Chem Sci. 2018 Feb 9;9(14):3503–7. doi: 10.1039/c7sc04331g (PMC5934698; doi:10.1039/c7sc04331g)
Supplement: Supplementary file 1 [file SC-009-C7SC04331G-s001.pdf]

Electronic Supplementary Material (ESI) for Chemical Science.
This journal is © The Royal Society of Chemistry 2018

On-chip measurements of protein unfolding from direct observations of micron-scale diffusion

Yuewen Zhang[a], Emma V. Yates[a], Liu Hong [a b], Kadi L. Saar[a], Georg Meisl[a], Christopher M. Dobson[a *], and Tuomas P. J. Knowles[ac *]

[a *] *Department of Chemistry, University of Cambridge, Lensfield Road, Cambridge, CB2 1EW, United Kingdom. E-mail: cmd44@cam.ac.uk; tpjk2@cam.ac.uk. Phone: +44 (0)1223 336344 .*

[b] *Zhou Pei-Yuan Center for Applied Mathematics, Tsinghua University, Beijing, 10084, P.R. China*

[c] *Cavendish Laboratory, University of Cambridge, J J Thomson Avenue, Cambridge, CB3 0HE, United Kingdom*

S.1 Experimental section

S.1.1 Materials

Bovine serum albumin (BSA), (#A7906), sodium bicarbonate (#8875), sodium carbonate (#S2127), ortho-phthalaldehyde (OPA), (#79760), sodium phosphate dibasic (#S0876) and sodium phosphate monobasic (#S5011), sodium dodecyl sulfate (SDS), (#71725) were obtained from Sigma-Aldrich. β-mercaptoethanol (BME), (#35602) was obtained from Thermo Scientific (Leicestershire, UK). All solutions were prepared using ultrapure water and filtered.

The standard labelling solution was 12 mM OPA, 18 mM BME, and 20% w/v SDS in 200 mM sodium carbonate buffer, pH 10.5[1]. The labelling solution was protected from light, stored at room temperature and used within 3 days of preparation.

S.1.2 Measurement of protein concentration

BSA was dissolved in 5 mM sodium phosphate buffer with different pH values. The pH value was observed not to change substantially after addition of the small concentration of BSA. The concentrations of BSA were measured using standard protein A280 settings on Nanodrop spectrophotometer. The molar extension coefficient (ε) for BSA is 43,824 $M^{-1}cm^{-1}$.

The hydrodynamic radius is only affect when the concentration of the protein reaches a level which is high enough to affect viscosity. In the present paper, the measurements are taken at concentrations below this value, and the changes in viscosity have been characterised previously[2].

S.1.3 Circular Dichroism (CD) experiment and data analysis

Samples for the CD experiment were prepared by incubating 3 μM BSA in 5 mM sodium phosphate buffer with different pH values at room temperature for at least 2 h. Far-UV (spectra between 250 nm to 200 nm) were recorded on JASCO J-810 equipped with a Peltier thermally controlled cuvette holder at 25 °C. A 1 mm path length quartz cuvette was used, and CD spectra were collected by averaging five individual recordings with the data pitch 0.5 nm, bandwidth 1 nm, scanning speed 50 nm/min and response time 1 s. Each CD measurement was repeated three times. The data was converted to the molar ellipticity ([θ]) in deg cm^{2}dmol^{-1} by using the formula[3]

$$[\theta] = \frac{\text{millidegrees} \times \text{mean residue weight}}{\text{pathlength in millimeters} \times \text{concentration in mg/ml}} \tag{1}$$

S.1.4 Microfluidic device fabrication

The microfluidic device was fabricated in polydimethylsiloxane (PDMS) based on standard soft-lithography techniques[4]. In brief, the device height was set by spin coating negative epoxy photoresist (MicroChem, product SU-8 3025) onto a silicon wafer. The silicon master with positive feature was prepared by blocking part of the photoresist with the acetate mask and cross-linking the exposed region with the exposed UV light. Propylene glycol mono methyl ether acetate (PGMEA) developer (MicroChem) was used to remove uncross-linked polymer. Then, the microfluidic device was cast in PDMS, which was prepared with a 10:1 weight ratio of PDMS elastomer and curing agent (Dow Corning, product 184). Around 1 mg/mL black carbon nanopowder (Sigma, product 633100) was added to the elastomer/curing agent to reduce the noise during image acquisition. The mixture was then poured onto the master and bubbles were removed by vacuum desiccation for 30 minutes. Finally the device was baked at 70 °C for around 1.5 h. After the device cooled, the device was cut out and holes were punched with 0.75 mm diameter Harris Uni-Core punchers. All debris were removed with selotape and sonicated with isopropanol (IPA). It is important to remove all residual IPA before the bonding step, thus the device was dried with nitrogen and baked for 10 minutes.

The device was bonded to Thermo Scientific 76 x 26 mm glass slides (catalog 8037) using an Electronic Diener Femto Plasma bonder. To ensure that the channels of the device were hydrophilic, the bonding first involved a 10 s generation of oxygen plasma. After generation, the device was put in contact with the glass slide, and then baked for 10 minutes to form a completely sealed device. Then, the sealed device was exposed to oxygen plasma for 500 s. In order to prevent hydrophobic recovery[5], the device was immediately filled with water after the bonding step by using portex 0.38 mm internal diameter, 1.09 mm external diameter tubing, connected to 1 mL plastic Air-Tite syringes. The inlets and outlet were blocked with water-filled gel loading tips. Devices stored in this way can be used for at least one week.

S.1.5 Microfluidic experiments

The devices were filled with buffer using 1 mL plastic syringe connected with 27-gauge needle using portex tubing. Samples and buffer were filtered by a 0.22 μm syringe filter (Millipore) immediately before use, which can reduce the chance of device clogging. Reagents were loaded into the device via gel-loading tips; depending on the reagents 10 μL-200 μL can be loaded. The flow was controlled by

neMESYS syringe pump. First 20 μL fluid with the 300 μL/h flow rate was withdrawn to eliminate potential inlet cross flow from the loading step. After that, a flow rate of 33.3 μL/h was used and allowed to equilibrate for approximately 18 minutes before image acquisition. Images were illuminated with 365 nm Cairn OptoLED (Photometrics) equipped with a Chromo 49000 DAPI filter and a CCD camera (Photometrics Evolve 512) for the fluorescent images. Different objectives (2.5X, 5X, 10X, and 20X) were used, exposure times of images between 10 ms to 10 s were selected, and 60 images were averaged during each acquisition. For each set of measurements, a background image was taken to account for the minimal fluorescence of the unreacted dye, and a flatfield background image was also acquired. Our system is at steady state, so actually the measurement time is limited only by the image acquisition time required to achieve sufficient signal, usually around 1 s. Measurements were taken in dark environment and the temperature was controlled at 25 °C.
The procedures outlined in S.1.4 and S.1.5 are similar to what we have reported in our previous work[1].

S.2 Labelling interactions on the microfluidic diffusional sizing (MDS) device

The hydrodynamic radius (R_h) of BSA is calculated based on fluorescence intensity recorded in the observation region (Fig. 1). Fluorescence is generated by a reaction between OPA and primary amines. Primary amine groups, such as lysine groups and the protein N-terminus, react with the OPA dialdehyde in the presence of the thiol group in BME to forms a conjugated pyrrole ring, resulting in the formation of a substituted isoindole, and fluorescence in the blue region of the spectrum is generated[6,7] (Fig.S1). High concentrations of SDS were added to ensure all primary amines were exposed and available for reaction[1,8].

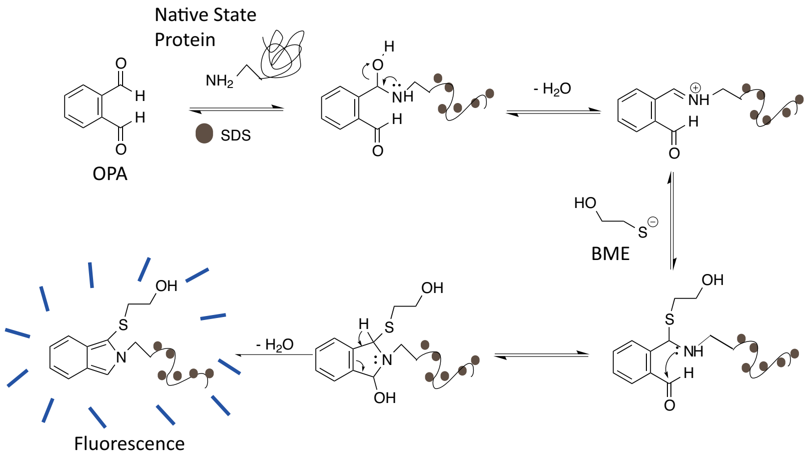

Figure S1: The process of fluorogenic labelling. Native proteins are denatured with SDS to expose all the primary amine groups. OPA is not fluorescent until it reacts with a primary amine to form a substituted isoindole. The fluorescence is formed in the blue region of the spectrum.

S.3 The R_h of BSA in buffers with different pH

The BSA stability was studied by preparing protein samples in buffers of varying pH. The changed R_h of BSA (Table S1) was calculated based on the fluorescence intensity generated by on-chip labelling.

Buffer with different pH	R_h of BSA (nm) *
1.2	8.40 ± 0.16
2.0	7.73 ± 0.21
2.5	6.25 ± 0.20
2.7	5.93 ± 0.31
3.3	5.17 ± 0.90
3.7	4.66 ± 0.40
4.3	3.30 ± 0.52
5.0	3.31± 0.62
6.0	3.78 ± 0.18
7.0	3.60 ± 0.41
8.0	3.80 ± 0.26
9.0	3.50 ± 0.37
10.2	3.34 ± 0.42

Table S1: The R_h of BSA in buffer with different pH.

* R_h is measured with the MDS device at 25 °C in a dark environment. All the measurements were done three times and the error bars represent the standard deviation among independent replicates.

S.4 Simulation the behaviour of different populations of folded and unfolded BSA

The movement of 10^6 particles in various compositions of folded (3.4 nm) and unfolded (8.4 nm) BSA was simulated in a rectangular channel of 200 μm width, 25 μm height and 17000 μm length at a flow rate of 25 μL/h[9]. The simulations are based on solving the Langevin equation describing diffusion advection behaviour[9–12]. For each ratio of folded and unfolded BSA, the diffusivity simulated as follows: one with half of the channel filled with the protein molecules and the other with the full channel filled in order to match the experimental protocol. By integrating the particle distribution across the rectangular channel at the end of the diffusion channel (t_D, Fig. 3b), we obtain the number of molecules that had diffused far enough to enter the fluid stream that flows into the labelling region of the device. From these profiles for each of the simulations, we extracted the number of molecules that had diffused far enough to enter the fluid stream that flows into the labelling region of the device (Fig. 3b dotted line). By comparing the relative intensities of the two simulations, we constructed a calibration curve that linked the recorded fluorescence intensity ratios to the fraction of folded and unfolded protein molecules (Fig. 3c). Software we used for the simulations is C^{++}. Further details about the simulation could be found in Ref. 8, published by our group recently.

S.5 The R_h and number of residues for a variety of folded and unfolded proteins

The value of folded and unfolded BSA is measured via the MDS device. The data of BSA was fitted (Fig. 2) to the literature values for R_h and number of residues for a variety of different proteins[13].

Proteins	R_h (Å) of folded proteins	R_h (Å) of unfolded proteins	Number of residues	Desaturation conditions	Reference
BSA	36 ± 4.1	84 ± 1.6	589	pH 1.2	
bovine ubiquitin	13.2	26.3	76	5M urea	Ref. 14
horse ferricytochrome c	13.5 ± 0.1	32.4 ± 1.6	104	4M GuHCl	Ref. 15
horse ferricytochrome c	13.5 ± 0.1	46 ± 0.5	104	pH 2.3	Ref. 16
staphylococcal nuclease	15.9 ± 0.2	35	149	5M urea	Ref. 17
horse myoglobin	17.5 ± 0.1	35.8 ± 1	153	in GuHCl	Ref. 18
bovine carbonic anhydrase B	19 ± 2	59 ± 2	259	4.5M GuHCl	Ref. 19
yeast phosphoglycerate kinase	23.4 ± 0.2	84 ± 1.6	589	4M GuHCl	Ref. 20

Table S2: The R_h and number of residues of several folded and unfolded proteins.

S.6 Determination of the fraction of α-helix

The normalized fraction of α-helices (with respect to maximal amount of α-helices of BSA) can be extracted from the mean residue ellipticity measured by far-UV CD at 222 nm, as

$$\mathrm{f}_\alpha = \left(\frac{\Theta_{222}^{\mathrm{obs}} - \Theta_{222}^{\mathrm{min}}}{\Theta_{222}^{\mathrm{max}} - \Theta_{222}^{\mathrm{min}}} \right) \times \left(\mathrm{f}_\alpha^{\mathrm{N}} - \mathrm{f}_\alpha^{\mathrm{U}} \right) + \mathrm{f}_\alpha^{\mathrm{U}} \tag{2}$$

where Θ_{222}^{obs}, Θ_{222}^{max} and Θ_{222}^{min} are the observed, maximal and minimal mean residue ellipticity at 222 nm respectively. $\mathrm{f}_\alpha^{\mathrm{U}}$ and $\mathrm{f}_\alpha^{\mathrm{N}}$ are the fraction of α-helix in the unfolded and folded proteins. Here a linear relationship is assumed between the ellipticity and secondary structure content.

To explain the dependence of α-helix content on pH conditions, we notice the fact that, due to the electrostatic repulsion, the α-helix structure becomes unstable in the presence of a high concentration of hydrogen ions. The unfolding of BSA through residue protonation at low pH values is a consequence of this mechanism. Since on average 73% of residues are in the helical form[21], a good correlation could be assumed between the fraction of α-helix and the degree of protein folding $f_N \in [0,1]$,

$$\mathrm{f}_\alpha = \mathrm{f}_\mathrm{N}\mathrm{f}_\alpha^{\mathrm{N}} + (1 - \mathrm{f}_\mathrm{N})\mathrm{f}_\alpha^{\mathrm{U}} \tag{3}$$

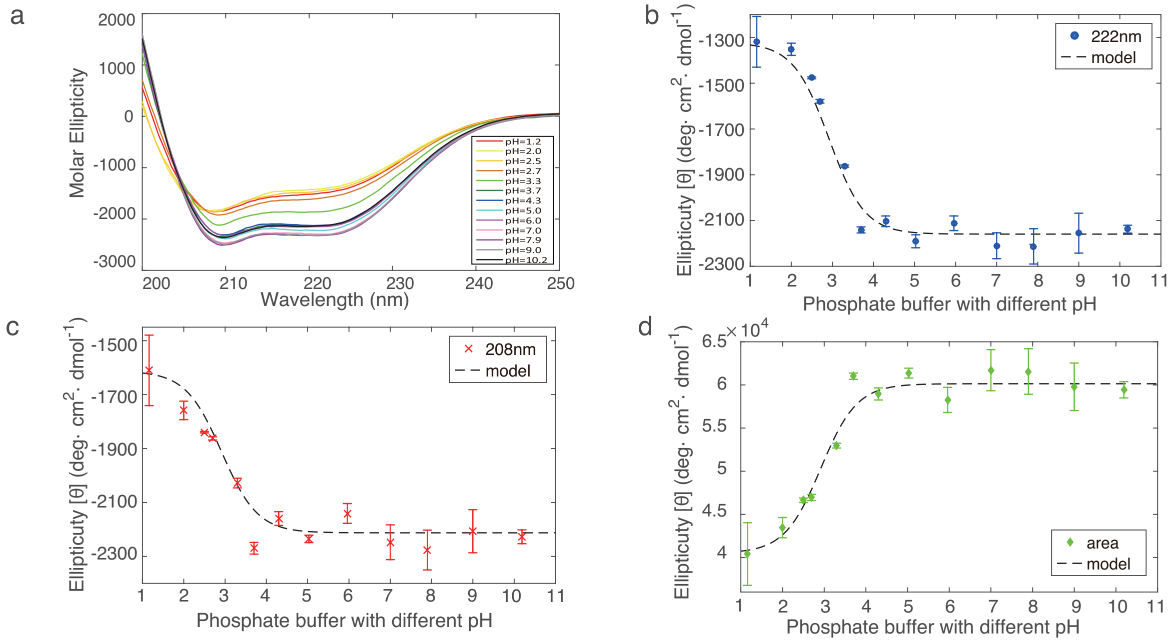

Figure S2: Effect of the pH value on the secondary structure of BSA (3 μM) in sodium phosphate (5 mM) buffer using far-UV CD (200 nm - 250 nm). Based on the far-UV CD spectra (a), the molar ellipticity of BSA at 222 nm (b), 208 nm (c) and total area between 200-250 nm (d) are calculated. All the measurements were done three times and error bars represent the standard deviation among independent replicates.

S.7 Model for low pH-induced protein unfolding

The acid induced unfolding of a protein molecule driven by the protonation of a single residue with an ionizable side group can be captured through the thermodynamic cycle[22,23]

$$\begin{array}{ccc} \mathrm{N} & \underset{+\mathrm{H}}{\overset{\mathrm{K}_a}{\rightleftharpoons}} & \mathrm{NH} \\ K_N \upharpoonleft\downharpoonright & & \tilde{K}_N \upharpoonleft\downharpoonright \\ \mathrm{U} & \underset{+\mathrm{H}}{\overset{\tilde{\mathrm{K}}_a}{\rightleftharpoons}} & \mathrm{UH} \end{array} \tag{4}$$

in which H, U, N, UH and NH represent protons, unfolded and folded states, the protonated unfolded and folded states of BSA respectively. K_N, $\tilde{\mathrm{K}}_N$ are the equilibrium constants for protein folding and unfolding, and pK_a, $\mathrm{p}\tilde{\mathrm{K}}_a$ are the logarithm acid dissociation constants for folded and unfolded BSA.

In the equilibrium state, we have

$$\mathrm{K}_N = \frac{[\mathrm{N}]}{[\mathrm{U}]}, \quad \mathrm{K}_a = \frac{[\mathrm{H}^+][\mathrm{N}]}{[\mathrm{NH}]}, \quad \tilde{\mathrm{K}}_a = \frac{[\mathrm{H}^+][\mathrm{U}]}{[\mathrm{UH}]}, \quad \tilde{\mathrm{K}}_N = \frac{[\mathrm{NH}]}{[\mathrm{UH}]} \tag{5}$$

Then the ratio between folded and unfolded proteins is given through

$$\frac{\mathrm{f_N}}{1-\mathrm{f_N}} = \frac{[\mathrm{N}]+[\mathrm{NH}]}{[\mathrm{U}]+[\mathrm{UH}]} = \frac{[\mathrm{N}]}{[\mathrm{U}]}\left(\frac{1+[\mathrm{H}^+]/\mathrm{K}_a}{1+[\mathrm{H}^+]/\tilde{\mathrm{K}}_a}\right) = \mathrm{K}_N\left(\frac{1+10^{\mathrm{pK}_a-\mathrm{pH}}}{1+10^{\mathrm{p}\tilde{\mathrm{K}}_a-\mathrm{pH}}}\right), \tag{6}$$

where we have used the definition of pH and pK_a values, pH$=-\log_{10}[H^+]$, p$K_a = -\log_{10} K_a$ and p$\tilde{K}_a = -\log_{10}\tilde{K}_a$. If we make the assumption that p$K_a \ll$ pH $\ll p\tilde{K}_a$ this simplifies to the expression given in the main text.

By assuming the titration sites are independent, this model can be generalized to include multiple titration sites:

$$\begin{aligned} \frac{f_N}{1-f_N} &= \frac{\sum_{i=0}^{m}[[NH]_i]}{\sum_{i=0}^{m}[[UH]_i]} = \frac{[N]}{[U]}\left(\frac{1+[H^+]/K_a^{(1)}}{1+[H^+]/\tilde{K}_a^{(1)}}\right)\left(\frac{1+[H^+]/K_a^{(2)}}{1+[H^+]/\tilde{K}_a^{(2)}}\right)\cdots\left(\frac{1+[H^+]/K_a^{(m)}}{1+[H^+]/\tilde{K}_a^{(m)}}\right) \\ &= K_N\left(\frac{1+10^{pK_a^{(1)}-pH}}{1+10^{p\tilde{K}_a^{(1)}-pH}}\right)\left(\frac{1+10^{pK_a^{(2)}-pH}}{1+10^{p\tilde{K}_a^{(2)}-pH}}\right)\cdots\left(\frac{1+10^{pK_a^{(m)}-pH}}{1+10^{p\tilde{K}_a^{(m)}-pH}}\right), \end{aligned} \quad (7)$$

where UH_i and NH_i denote the concentrations of protonated unfolded and folded BSA with i protons. pK_a^i and p$\tilde{K}_a^i$ are the logarithm equilibrium constants for the deprotonation of titration site i in folded and unfolded BSA respectively. p$K_a^i = -\log_{10} K_a^i$ and p$\tilde{K}_a^i = -\log_{10}\tilde{K}_a^i$ are defined accordingly. As a consequence, the difference of free energy between folded and unfolded states under different pH conditions is

$$\triangle G(\mathrm{pH}) = \triangle G^{\ominus} - k_B T \sum_{i=1}^{m} \ln\left(\frac{1+10^{pK_a^{(i)}-pH}}{1+10^{p\tilde{K}_a^{(i)}-pH}}\right), \quad (8)$$

where $\triangle G^{\ominus} = -k_B T \ln K_N$ is the free energy of folding at pH=7.

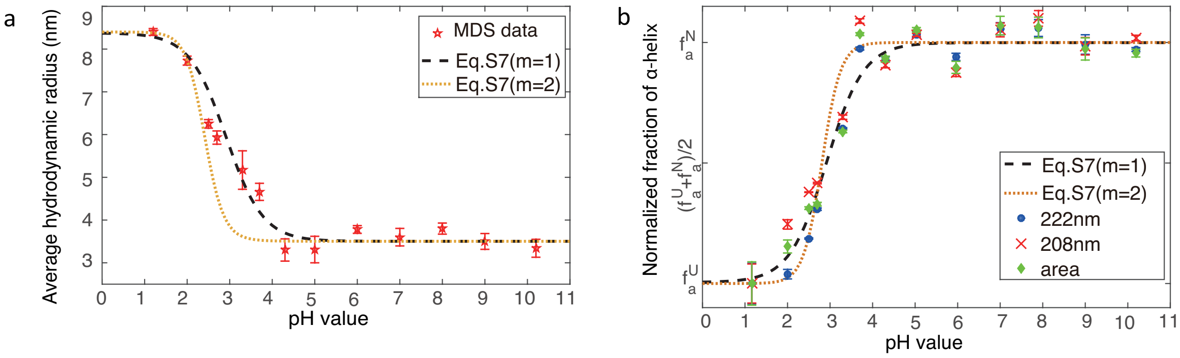

Figure S3: (a): Rh value of BSA in buffer solutions with varying pH values. The Rh values were measured by using the microfluidic diffusional sizing device. Fitting was performed with Eq. S7 and linear interpolation $R_h = (R_h^{max} - R_h^{min})\cdot(1-f_N) + R_h^{min}$, values of R_h^{max}=8.4 nm and R_h^{min}=3.5 nm were determined directly from the data. (b): The renormalized fraction of α-helical derived from the molar ellipticity at 222 nm, 208 nm and total area are calculated using Eq. S3. Predictions based on one titration site (His241) and two other titration sites (here we tried any two combinations of Asp, Glu and His) were performed according to Eq. S7 and $f_\alpha = f_\alpha^U + (f_\alpha^N - f_\alpha^U)\cdot f_N$, f_α^U and f_α^N are the fraction of α-helix in the unfolded and native proteins.

S.8 Prediction of pK_a values for His, Asp and Glu

In principle only the side-chain protonation of His, Asp and Glu is related to BSA unfolding at low pH values. In the main text, the difference of pK_a values of these three residues in the folded state are calculated (Fig. 5) based on the DEPTH web server[24]. Here we separately show the pK_a and $p\tilde{K}_a$ values of His, Glu and Asp residues respectively.

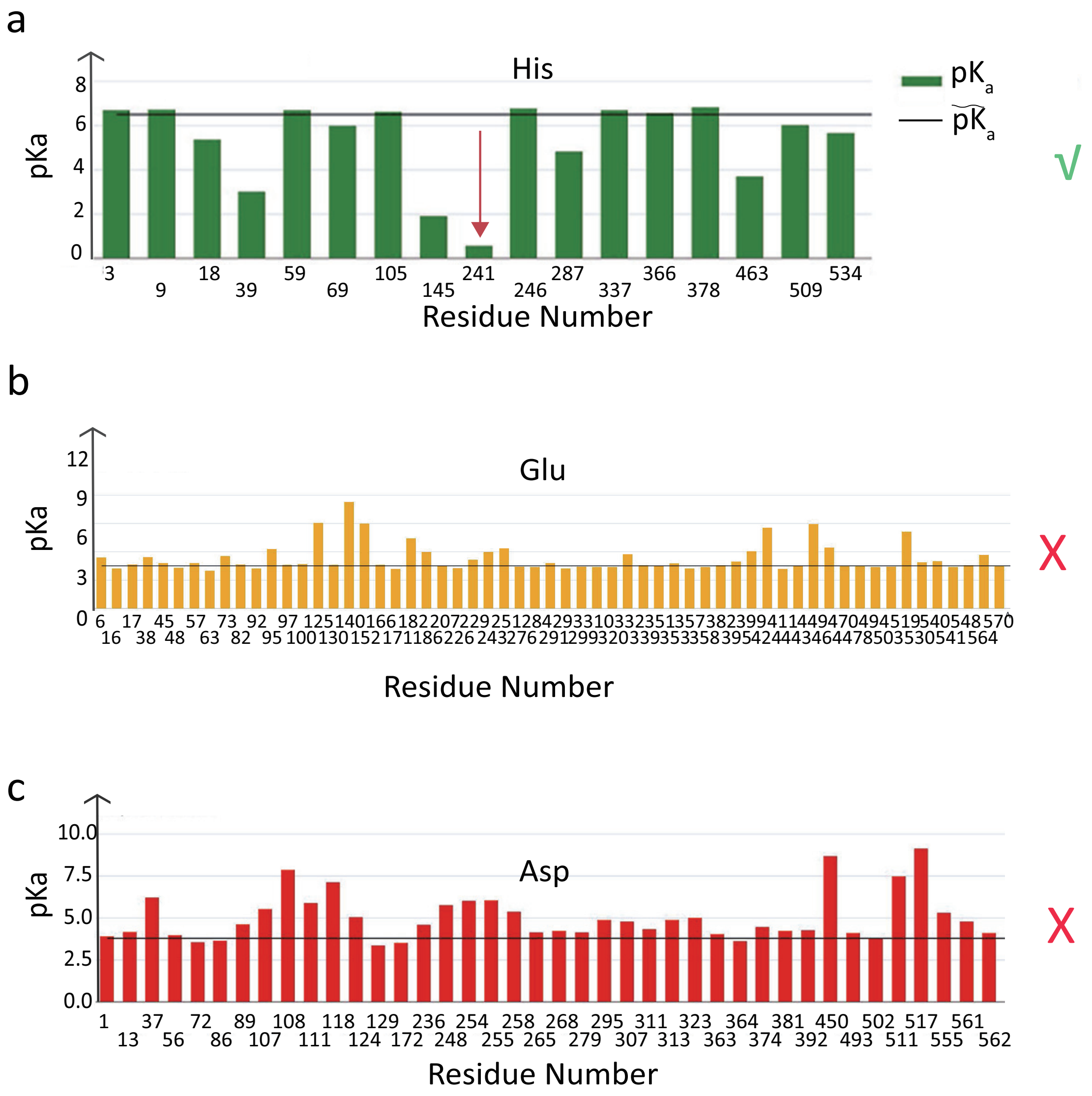

Figure S4: Predicted pK_a value of His, Glu and Asp residues based on the DEPTH web server. The predicted pK_a value in the folded state of His, Glu and Asp is shown with a green, yellow and red bar respectively, while that in the unfolded state is given by the solid black line. (a), The pK_a value of His residues in the folded BSA protein. One key titration site His241(red arrow) is highlighted with $p\tilde{K}_a = 6.04$ and $pK_a = 0.6$. (b) and (c), pK_a values for Glu and Asp residues in the folded and unfolded states do not meet our requirement on the selection of potential titration sites (details in the main text).

References

[1] E. V. Yates, T. Müller, L. Rajah, E. J. D. Genst, P. Arosio, S. Linse, M. Vendruscolo, C. M. Dobson and T. P. J. Knowles, *Nat. Chem.*, 2015, **7**, 802–809.

[2] P. Arosio, K. Hu, F. A. Aprile, T. Müller and T. P. Knowles, *Anal. Chem.*, 2016, **88**, 3488–3493.

[3] N. J. Greenfield, *Nat. Protoc.*, 2006, **1**, 2876–2890.

[4] J. C. Mcdonald, D. C. Duffy, J. R. Anderson and D. T. Chiu, *Electrophoresis*, 2000, **21**, 27–40.

[5] S. H. Tan, N. Nguyen, Y. C. Chua and T. G. Kang, *Biomicrofluidics*, 2010, **4**, 032204.

[6] S. S. Simons and D. F. Johnson, *J. Am. Chem. Soc.*, 1976, **98**, 7098–7099.

[7] L. A. Sternson, J. F. Stobaugh and A. J. Repta, *Anal. Biochem.*, 1985, **144**, 233–246.

[8] D. Otzen, *Biochim. Biophys. Acta*, 2011, **1814**, 562–591.

[9] T. Müller, P. Arosio, L. Rajah, S. I. Cohen, E. V. Yates, M. Vendruscolo, C. M. Dobson and T. P. Knowles, *INT J NONLIN SCI NUM*, 2016, **17**, 175–183.

[10] T. Su, J. Lu, R. Thomas, Z. Cui and J. Penfold, *J. Phys. Chem. B*, 1998, **102**, 8100–8108.

[11] J. P. Brody and P. Yager, *Sens. Actuat. A*, 1997, **58**, 13–18.

[12] B. H. Weigl and P. Yager, *Science*, 1999, **283**, 346–347.

[13] D. K. Wilkins, S. B. Grimshaw, V. Receveur, C. M. Dobson, J. A. Jones and L. J. Smith, *Biochemistry*, 1999, **38**, 16424–16431.

[14] Y. O. Kamatari, S. Ohji, T. Konno, Y. Seki, K. Soda, M. Kataoka and K. Akasaka, *Protein Sci*, 1999, **8**, 873–882.

[15] M. Kataoka, Y. Hagihara, K. Mihara and Y. Goto, *J. Mol. Biol.*, 1993, **229**, 591–596.

[16] G. Damaschun, H. Damaschun, K. Gast, C. Gernat and D. Zirwer, *Biochim. Biophys. Acta*, 1991, **1078**, 289–295.

[17] J. M. Flanagan, M. Kataoka, D. Shortle and D. M. Engelman, *PNAS*, 1992, **89**, 748–752.

[18] M. Kataoka, I. Nishii, T. Fujisawa, T. Ueki, F. Tokunaga and Y. Goto, *J. Mol. Biol.*, 1995, **249**, 215–228.

[19] G. V. Semisotnov, H. Kihara, N. V. Kotova, K. Kimura, Y. Amemiya, K. Wakabayashi, I. N. Serdyuk, A. A. Timchenko, K. Chiba, K. Nikaido *et al.*, *J. Mol. Biol.*, 1996, **262**, 559–574.

[20] V. Receveur, D. Durand, M. Desmadril and P. Calmettes, *FEBS Lett.*, 1998, **426**, 57–61.

[21] F. Putnam, *The Plasma Proteins V4: Structure, Function, and Genetic Control*, Elsevier, 2012.

[22] D. E. Anderson, W. J. Becktel and F. W. Dahlquist, *Biochemistry*, 1990, **29**, 2403–2408.

[23] S. Walter, B. Hubner, U. Hahn and F. X. Schmid, *J. Mol. Biol.*, 1995, **252**, 133–143.

[24] K. P. Tan, T. B. Nguyen, S. Patel, R. Varadarajan and M. S. Madhusudhan, *Nucleic Acids Res*, 2013, **41**, W314–W321.
